# Dissecting the Roles of PDCD4 in Breast Cancer

**DOI:** 10.3389/fonc.2022.855807

**Published:** 2022-06-20

**Authors:** Qian Cai, Hsin-Sheng Yang, Yi-Chen Li, Jiang Zhu

**Affiliations:** ^1^Department of Geriatric Medicine, Qilu Hospital of Shandong University, Jinan, China; ^2^Key Laboratory of Cardiovasular Proteomics of Shandong Province, Qilu Hospital of Shandong University, Jinan, China; ^3^Department of Toxicology and Cancer Biology, Collage of Medicine, University of Kentucky, Lexington, KY, United States; ^4^Department of Breast Surgery, General Surgery, Qilu Hospital of Shandong University, Jinan, China

**Keywords:** PDCD4, breast cancer, translational control, drug resistance, tumor suppressor

## Abstract

The human programmed cell death 4 (*PDCD4*) gene was mapped at chromosome 10q24 and encodes the PDCD4 protein comprised of 469 amino acids. PDCD4 inhibits protein translation PDCD4 inhibits protein translation to suppress tumor progression, and its expression is frequently decreased in breast cancer. PDCD4 blocks translation initiation complex by binding eIF4A *via* MA-3 domains or by directly binding 5’ mRNA internal ribosome entry sites with an RNA binding domain to suppress breast cancer progression and proliferation. Numerous regulators and biological processes including non-coding RNAs, proteasomes, estrogen, natural compounds and inflammation control PDCD4 expression in breast cancer. Loss of PDCD4 expression is also responsible for drug resistance in breast cancer. HER2 activation downregulates PDCD4 expression by activating MAPK, AKT, and miR-21 in aromatase inhibitor-resistant breast cancer cells. Moreover, modulating the microRNA/PDCD4 axis maybe an effective strategy for overcoming chemoresistance in breast cancer. Down-regulation of PDCD4 is significantly associated with short overall survival of patients, which suggests that PDCD4 may be an independent prognostic marker for breast cancer.

## Introduction

Programmed cell death 4 (PDCD4) was first isolated as an antigen in proliferating cells that is bound by the monoclonal antibody Pr-28. Subsequently, the *PDCD4* gene was identified in different species, including humans (1, 2), rats (3), and chicken (4). The human PDCD4 protein is comprised of 469 amino acids, and the protein sequence is a highly conserved from *Drosophila* to humans. The mouse and human PDCD4 proteins share approximately 92% identity in amino acid sequence. The human *PDCD4* gene was mapped at chromosome 10q24 (5). PDCD4 has been demonstrated to be a protein translation inhibitor and a tumor suppressor, and consistently its expression frequently decreased in numerous types of malignancies including breast, colon, liver, lung, pancreatic, and skin cancers (6, 7). Loss of PDCD4 expression is associated with poor prognosis of patients with breast cancer (8, 9). In our review, we searched literatures in pubmed with the keywords PDCD4 and breast cancer in all fields. According to the contents of the target references, the article will focus on the function, regulation, and prognostic value of PDCD4 in breast cancer as well as its role in drug resistance.

## PDCD4 Functions

### Inhibition of Protein Translation

**Figure 1:**
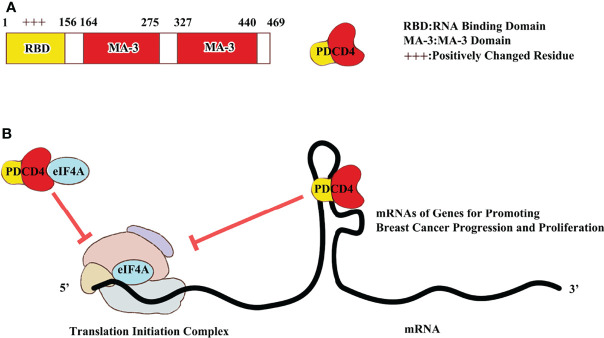
PDCD4 protein structure and mechanism to regulate translation initiation. **(A)** PDCD4 protein is 469 amino acid length with one RNA binding domain and two MA-3 domains. **(B)** PDCD4 protein could block translation initiation by either binding IRES in 5’ UTR or eIF4A in 48S translation initiation complex.

Using yeast-two hybrid, PDCD4 was identified as a binding partner of translation initiation factor 4A (eIF4A) (10). The PDCD4-eIF4A interaction was further confirmed by mutation and crystallography (11–13). Crystallography of PDCD4-eIF4A showed that one molecule of PDCD4 interacts with two molecules of eIF4A through two MA-3 domains in PDCD4. The Glu210, Glu249, and Asp253 residues in the N-terminal segment of the MA-3 domain and the Glu373, Arg414, and Asp418 residues in the C-terminal segment form the ionic interaction with Arg110 and Arg161 of eIF4A (11, 12). Mutation of Glu249 to Lys, Asp253 to Ala, Asp414 to Lys and/or Asp418 to Ala in PDCD4 abolish its ability to bind eIF4A and result in loss of the ability to inhibit protein translation (13). This indicates that binding with eIF4A is required for the inhibition of translation by PDCD4. How does PDCD4-eIF4A binding inhibits protein translation? One possible mechanism is that PDCD4 inhibits protein translation through suppression of eIF4A’s helicase activity (10). eIF4A is an ATP-dependent RNA helicase. The function of eIF4A in protein translation initiation is to unwind the structured mRNA, allowing the translation initiation complex scan the mRNA from 5’ to 3’ end to locate the translation initiation codon (14). Therefore, inhibition of eIF4A activity by PDCD4 is expected to suppress protein translation, especially translation of mRNAs with complexly structured 5’untranslated regions (5’UTRs). PDCD4 was initially demonstrated to preferentially suppress translation of a luciferase mRNA with a stem loop-structured 5’UTR with free energy of -44.8 kcal/mol (13). This scenario was further confirmed by identification of the stress-activated-protein kinase interacting protein 1 (Sin1) and ribosomal S6 kinase 1 (S6K1) as PDCD4 targets, whose mRNAs have 5’UTRs that form secondary structures with free energies of -145 and -82.30 kcal/mol, respectively (15, 16). In agreement with inhibition of eIF4A by PDCD4, cells treated with an eIF4A inhibitor, silvestrol, had reduced the Sin1 protein abundance (16). It seems that the mRNAs that have 5’UTRs with a free energy greater than -44.8 kcal/mol are susceptible to translation inhibition by PDCD4. However, this notion requires identification and analysis of additional PDCD4 targets. In addition to inhibiting eIF4A activity, PDCD4 may inhibit translation of anti-apoptotic proteins such as Bcl-xl and XIAP by directly binding with their mRNAs at internal ribosome entry sites to block the translation initiation complex formation (17, 18). The mechanisms of translation inhibiton by PDCD4 are summarized in **Figure 1**.

### Suppression of Tumorigenesis

PDCD4 was initially suggested to be a tumor suppressor in transformation-resistant JB6 cells because the down-regulation of PDCD4 expression by antisense RNA resulted in a gain of transformation-susceptible phenotype (19). Conversely, overexpression of PDCD4 cDNA in the transformation-susceptible JB6 cells endowed resistance to tumor promoter-induced transformation (20). Mouse epidermal JB6 cells consist of three variants including transformation-susceptible, transformation-resistant, and transformed cells. Transformation-susceptible JB6 cells are sensitive to tumor promoter-induced transformation such as that induced by 12-O-tetradecanoylphorbol-13-acetate (TPA) treatment. By contrast, transformation-resistant JB6 cells are resistant to TPA-induced transformation. Later, several additional studies demonstrated that PDCD4 not only inhibits tumor promoter-induced transformation but also suppresses cancer cell proliferation, migration, invasion, and metastasis (for detail please see reviews (21, 22)). In breast cancer, PDCD4 has been shown to be a pro-apoptotic factor and inhibits cell growth (23, 24). Overexpression of PDCD4 reduces the rate of proliferation and promotes apoptosis in T-47D, MDA-MB-231, and MCF-7 breast cancer cells (25, 26). Conversely, down-regulation or repression of PDCD4 decreases apoptosis (27, 28). In addition to suppressing proliferation and cell growth, several studies also demonstrated that PDCD4 attenuates tumor invasion and metastasis. Immunohistochemical studies showed that PDCD4 expression was only slightly decreased in *in situ* ductal carcinoma samples but was markedly decreased in invasive ductal carcinoma samples (29). In addition, reverse phase protein arrays, a new technique to study the functional proteome in non-microdissected breast tumors (30), showed that loss of PDCD4 expression was significantly associated with lymph node metastasis in HER2- and ER-positive invasive breast carcinomas (31). These findings suggest that loss of PDCD4 expression promotes breast cancer invasion. This notion is further supported by *in vitro* studies showing that knockdown of PDCD4 boosts invasion of T47D cells (32). Recently, PDCD4 overexpression was demonstrated to suppress cell cycle progression from the G1 phase to the G2 phase in triple negative breast cancer (TNBC) cell line (33). Although it is clear that PDCD4 suppresses tumorigenesis in breast cancer, the underlying mechanism is still not fully understood. For example, PDCD4 inhibits invasion in breast cancer cells by reduction of inhibitors of metalloproteinases-2 (TIMP-2) expression (34, 35). TIMP-2 inhibits matrix metalloproteinase (MMP) activity to prevent degradation of the extracellular matrix and thereby impedes tumor cell migration and invasion (36). However, it is unknown of how PDCD4 inhibits TIMP-2 expression.

Currently, pieces of evidence suggest that PDCD4 may inhibit tumorigenesis through two possible mechanisms. As mentioned above, PDCD4 binds with eIF4A and inhibits its helicase activity. During translation initiation, eIF4A is required for the unwinding or mRNAs that have 5’UTRs with secondary structures. Usually, mRNAs with 5’UTRs that have secondary structures encode for growth factors, growth promotion genes, and proto-oncogenes (37). Thus, inhibition of eIF4A by PDCD4 is expected to cause suppression of cell proliferation, migration, and/or invasion. For example, PDCD4 inhibits Sin1 translation to attenuate cell invasion in colorectal carcinoma (16). Sin1 is a critical component of mammalian target of rapamycin complex 2 (mTORC2) that regulates Akt activation. Inhibition of Sin1 translation by PDCD4 results in reduction of Snail expression to suppress invasion *via* inactivation of Akt. In addition to the eIF4A-dependent mechanism, PDCD4 may repress tumorigenesis through an eIF4A-independent mechanism. Liwak et al. reported that PDCD4 directly binds with the mRNA of X-linked inhibitor of apoptosis (XIAP) at an internal ribosome binding site and inhibits XIAP translation by preventing the translation initiation complex formation (18). In addition, PDCD4 was reported to bind to rapamycin-insensitive companion of mTOR (Rictor), another key component of mTORC2, to repress mTORC2 activity (38), and this may regulate cell survival, migration, and invasion by inhibiting Akt phosphorylation. However, the role of PDCD4-Rictor binding in the suppression of tumorigenesis and the underlying mechanism needs further investigation.

## The Regulators of PDCD4 in Breast Cancer

### Non-Coding RNAs (microRNAs, lncRNAs and CircRNAs)

Non-coding RNAs (NcRNAs) include microRNAs (miRNAs), long non-coding RNAs (lncRNAs), and circular RNAs (CircRNAs). All of these have been reported to regulate the expression of PDCD4 and thereby control tumorigenesis in breast cancer.

miRNAs are small non-coding RNA molecules found in plants, animals and some viruses. They play critical roles in RNA silencing and post-transcriptional regulation of target genes. Among miRNAs, microRNA-21 (miR-21) was first identified to regulate PDCD4 expression (39). miR-21 is classified as an “oncomiR” and is commonly overexpressed in many types of solid cancers including breast cancer (40–42). Overexpression of miR-21 is implicated in various processes of tumorigenesis through the suppression of PDCD4 expression. These include the suppression of apoptosis (43, 44), promotion of cell proliferation and tumor growth (45, 46), and stimulation of invasion and metastasis (47, 48). Moreover, miR-21 significantly augmented PD-L1 expression and immune escape *via* PI3K/Akt pathway activation by targeting PDCD4 in breast cancer cells. According to this traits, Anti-PD-L1 antibody could enhance T cell immune responses and reduces resistance of breast cancer cells to radiotherapy in nude mice (49). Lu et al. found that the 3’-UTR of the PDCD4 mRNA contains a functional binding site for miR-21 (50). The PDCD4 mRNA level in MCF-7 cells did not change in the miR-21-transfected cells while the PDCD4 protein level decreased by approximately 50%, indicating that the reduction of PDCD4 protein is a result of post-transcriptional repression (50). Conversely. inhibition or down-regulation of miR-21 expression results in augmented PDCD4 expression (51). In addition to miR-21, miR-17-5p (52), miR-206 (53), miR-183-5p (54), miR-421 (28) and miR-424 (55) were reported to inhibit PDCD4 expression to influence the malignant behaviors of breast cancer cells (56).

lncRNAs are longer than 200 bp and comprise the most complex group of NcRNAs. lncRNAs contain miRNA binding sites and function as molecular sponges to effectively inhibit the expression of downstream genes targeted by miRNAs (57). A recent study showed that the expression of PDCD4-targeting lncRNA, PDCD4-AS1, is down-regulated during breast cancer progression, and its expression is positively correlated with PDCD4 level in breast cancer tissue (58, 59). Depletion of PDCD4-AS1 results in promotion of cell proliferation and migration in breast cancer cells. It is believed that PDCD4-AS1 stabilizes PDCD4 mRNA by forming an RNA duplex to attenuate the interaction between PDCD4 mRNA and RNA decay promoting factor HuR (60). Moreover, LncRNA GAS5 interacts with miR-21 *via* miRNA binding elements in breast cancer. Mediated by this sponge mechanism, GAS5 is involved in the upregulation of a number of mRNAs that encode tumor suppressor proteins such as PDCD4 (61). A similar mechanism that lncRNA SLC16A1-AS1 may serve as an internal sponge of miR-182 to augment PDCD4 has been reported recently. SLC16A1-AS1 is downregulated in TNBC and overexpression of SLC16A1-AS1 could suppress TNBC cell proliferation (33).

The circRNAs were initially identified from RNA viruses in 1976 and are connected at the 3’ and 5’ ends by exon or intron cyclization to form complete ring structures. CircRNAs have abundant binding sites for non-coding RNAs and thus act *via* absorption of non-coding RNAs like a sponge. CircRNAs affect the stability or the translation of target RNAs by competitively binding with miRNAs (62). For example, Hsa_circ_0053063 is a circRNA generated from several exons of hydroxyacyl CoA dehydrogenase trifunctional multienzyme complex subunit alpha (HADHA), and it inhibits cell viability, proliferation, and progression of breast cancer through stabilization of PDCD4 by targeting miR-330-3p (63). Similarly, Circ-NOL10 could suppress breast cancer carcinogenesis by upregulating PDCD4 *via* a circ-NOL10/miR-149-5p/miR-330-3p/miR-452-5p/PDCD4 pathway (64).

Exosomes are extracellular bilayer vesicles with diameters ranging from 30 to 100 nm that transfer a wealth of nucleic acids and proteins among cells under physiological and pathological conditions (65). Breast cancer cell-derived exosomes play an important role in promoting breast cancer bone metastasis, which is associated with the formation of a pre-metastatic niche *via* transferal of miR-21 to osteoclasts and consequent down-regulation of PDCD4 expression (66).

### Proteasomal Degradation

Proteasomes are part of a major mechanism by which cells regulate the concentration of particular proteins as well as degrade misfolded and unneeded proteins. Proteins are tagged for degradation by a small protein called ubiquitin. Compromised proteasome complex assembly leads to reduced proteolytic activity and the accumulation of damaged and misfolded proteins, and this contributes to the pathogenesis in many diseases (67). Proteasomal degradation is another important mechanism that regulates PDCD4 expression. In response to mitogens such as TPA, PDCD4 is rapidly phosphorylated at Ser67 by the 70-kDa ribosomal protein 6 kinase (*p70S6K*). The Ser67 phosphorylated PDCD4 is recognized and bound by the ubiquitin E3 ligase β-transducin repeat-containing 1 (β-TRCP-1). The subsequent ubiquitination targets PDCD4 for proteasomal degradation and ultimately promotes tumor formation (68). Down-regulation of p70S6K by *p70s6k* shRNA increased PDCD4 expression in MDA-MB-231 cells (69). In addition to p70S6K, it was reported that the other p70S6K family member, p90 ribosomal S6 kinase (p90RSK), was able to phosphorylate PDCD4 at Ser76 and lead to PDCD4 degradation in several TNBC cell lines (70). Conversely, ubiquitin-specific protease 4 (USP4) can increase PDCD4 expression through deubiquitination and subsequent inhibition of its degradation in MCF-7 cells (71). A recent study showed that the S-phase kinase-associated protein 2 (SKP2) promotes PDCD4 phosphorylation at Ser67 and subsequently its proteasomal degradation, resulting in the promotion of breast cancer cell proliferation (72). Interestingly, PDCD4 was also reported to inhibit IGF1R/IR inhibitor-induced phosphorylation of *p70S6K* (15, 73), which suggests a feedback loop regulation between PDCD4 and p70S6K.

### Inflammation

Accumulating evidence has shown that inflammation can promote all stages of tumorigenesis, including DNA damage, limitless replication, apoptosis evasion, sustained angiogenesis, self-sufficiency in growth signaling, insensitivity to anti-growth signaling, and tissue invasion/metastasis. An inflammatory tumor microenvironment may attenuate PDCD4 protein expression. For example, exposure of MCF-7 cells to conditioned medium from TPA-induced differentiated monocytes attenuate PDCD4 protein level. This conditioned medium contained proinflammatory cytokines such as tumor necrosis factor-alpha (TNF-α), interleukin-6 (IL-6), and IL-8, which stimulate PI3K–mTOR signaling pathway and thereby facilitate p70S6K-dependent proteasomal degradation of PDCD4 (74). In addition, activation of the Cyclooxygenase-2 (COX-2)/prostaglandin E2 (PGE2)/Interleukin-8 (IL-8) inflammatory pathway suppresses PDCD4 expression and promotes the invasion in MCF-7 cells (35). It also reported that proinflammatory cytokine transforming growth factor-β (TGF-β) stimulates miR-21 expression and thereby suppresses PDCD4 expression in MDA-MB-468 cells (75).

### Estrogen

Lifetime estrogen exposure is widely accepted as a major risk factor for breast cancer development (76). Several epidemiological surveys indicate that supplementation with exogenous estrogen or a high level of endogenous estrogen significantly increase the incidence of breast cancer (77). *In vitro* studies also showed that dimerized estrogen binds with estrogen receptor (ER) and promotes cell proliferation and invasion of breast tumor cells (78). Natural estrogens include estradiol (E2), estrone (E) and estrotriol (E3), among which estradiol is the most bioactive form involved in breast tumorigenesis (79). Anti-estrogens are a class of drugs that prevent estrogens like E2 from mediating their biological effects *in vivo*. They act by blocking ER or by suppressing estrogen production. ICI-182780, an artificial anti-estrogen drug also named fulvestrant, can increase PDCD4 protein expression in ER-positive T-47D cells (25, 80). Interestingly, E2 treatment increases PDCD4 expression in MCF-7 cells by down-regulation of miR-21 in an ER-dependent manner (81). Further research proved that overexpression of ERα may up-regulate miR-21 to suppress PDCD4 expression in breast cancer cells (82). This interesting phenomenon can be interpreted as follows: if ER density is not increased, breast cancer cells may overexpress PDCD4 in the face of high estrogen levels, and if ER density is increased rising sensitivity to estrogen may decrease PDCD4 expression and thereby promote metastasis.

### Natural Compounds

Sinomenine (Sino) is diffusely applied to heal rheumatoid arthritis and neuralgia. Gao et al. found that Sino exposure remarkably enhanced PDCD4 expression by inhibiting miR-29 in MDA-MB-231 cells (83). In addition, Gleditsia sinensis extract has been historically used in Chinese medicine and is considered one of the fundamental therapeutic herbs. The anti-breast cancer effect for Gleditsia sinensis probably results in upregulation of PDCD4 expression to promote cell apoptosis (84, 85). Curcumin is a constituent of the yellow powder extracted from the roots of Curcuma longa Linn. It is used to treat various diseases including hepatic disorders, anorexia, diabetic wounds, and rheumatism. Treatment with curcumin could down-regulates miR-21 expression and consistently up-regulates PDCD4 to promote apoptosis in breast cancer cell lines (86). Andrographolide is regarded as a “natural antibiotic” and is present in Andrographis paniculate (87), and it has shown to inhibit breast cancer growth and metastasis *in vivo* and to suppress cell proliferation, migration, and invasion *in vitro*. Mechanistically, andrographolide inhibited nuclear factor-kappa B (NF-κB) and elevates PDCD4 expression through suppression of miR-21-5p (88).

### Other Mechanisms to Regulate PDCD4 Expression

Retinoic acid receptors (RARs), which belong to steroid/thyroid hormone receptor gene family, are ligand-dependent transcription factors. They have been shown to inhibit the growth of breast cancer cells *in vitro* and *in vivo*. Afonja et al. treated T-47D and MCF-7 breast cancer cells with Am580 (a selective RARα agonist) and all-trans *retinoic acid* (ATRA, another RAR agonist derived from vitamin A retinoids) and found that PDCD4 expression was increased two- to five-fold. However, the detailed mechanism by which RAR agonists regulate PDCD4 expression is still unclear (25).

Human epidermal growth factor receptor-2 (HER2) is an oncogene, and its downstream signaling pathway plays important roles in the development and progression of certain aggressive types of breast cancer. HER2 antagonists (such as trastuzumab, pertuzumab and lapatinib) induce PDCD4 protein expression in T-47D cells, which express HER2 (25). Transfection of HER2 expression plasmid into MDA-MB-435 cells reduced PDCD4 expression by about 45% and concomitantly enhanced invasion and metastasis (89). Signal transducer and activator of transcription 3 (STAT3) was found to bind its response elements in the *HER2* promoter to upregulate *HER2* transcription in metastatic HER2-positive breast cancer. Moreover, STAT3 co-opts HER2 function by recruiting HER2 as its coactivator at the binding sites in the miR-21 promoter to enhance its expression, resulting in depression of PDCD4 (90).

The neuregulins (NRGs) comprise the largest subfamily of EGF-like polypeptide growth factors and act by binding to the ErbB/HER receptor tyrosine kinases. Breast cancer cells treated with NRGs have reduced phosphorylation of PDCD4 at Ser67, which results in increased PDCD4 stabilization due to avoidance of proteasomal degradation (91).

The chemical compound AC1MMYR2 (2,4-diamino-1, 3-diazinane-5-carbonitrile, also known as NSC211332) was identified as a specific small-molecule inhibitor of miR-21 that blocks the ability of Dicer to process pre-miR-21 to mature miR-21. AC1MMYR2 up-regulates the expression of PDCD4 to suppress proliferation and invasion in breast cancer (92).

In brief, various type of regulators control PDCD4 expression to influence hallmarks of breast cancer. Information on these regulatory factors is summarized in **Table 1**. Meanwhile, PDCD4 expression in different subtypes of breast cancer cell lines and tissues is summarized in **Table 2**.

**Table 1:** Information of PDCD4 regulators in breast cancer.

Name	Type	Regulatory Target or Pathways	Up/Down- Regulation	Ref No.
miR-21	NcRNA	3'-UTR of PDCD4	Down	37, 47
miR-17-5p	NcRNA	3'-UTR of PDCD4	Down	48
miR-206	NcRNA	3'-UTR of PDCD4	Down	49
miR-183-5p	NcRNA	3'-UTR of PDCD4	Down	50
miR-421	NcRNA	3'-UTR of PDCD4	Down	29
miR-424	NcRNA	3'-UTR of PDCD4	Down	51
PDCD4-AS1	NcRNA	mRNA of PDCD4	Up	52
LncRNA GAS5	NcRNA	miR-21	Up	54
SLC16A1-AS1	NcRNA	miR-182	Up	33
Hsa_circ_0053063	NcRNA	miR-330-3p	Up	62
Circ-NOL10	NcRNA	miR-149-5p/miR-330-3p/miR-452-5p	Up	59
p70S6K	Ribosomal protein	Ser^67^ phosphorylation	Down	57, 58
p90RSK/RSK	Ribosomal S6 kinase	S^76^ phosphorylation/MAPK pathway	Down	69, 70
USP4	Ubiquitin-specific protease	Deubiquitination	Up	71
SKP2	S-phase kinase	Ser^67^ phosphorylation/AKT pathway	Down	62
TNF-α/IL-6/IL-8	Proinflammatory cytokines	PI3K/mTOR/p70S6K pathway	Down	63
TGF-β	Proinflammatory cytokines	miR-21	Down	64
Cox-2	Cyclooxygenase	COX-2/PGE2/IL-8 inflammatory pathway	Down	33
Estradiol	Sexal Hormone	miR-21	Up	69
Fulvestrant	Antiestrogen drug	Estrogen	Up	26
Sinomenine	Natural compounds	miR-29	Up	72
*Gleditsia sinensis*	Natural compounds	miR-183	Up	73, 74
Curcumin	Natural compounds	miR-21	Up	75
Andrographolide	Natural compounds	NF-kB/miR-21-5p pathway	Up	77
Am580	Selective RARα-agonist	Unclear	Up	26
ATRA	Selective RARα-agonist	Unclear	Up	26
FOXP3	Tumor suppressor gene	Unclear	Up	25
Trastuzumab/Pertuzumab/	HER-2/neu antagonist	Unclear	Up	26
Lapatinib				
Stat3	Oncogene	Her-2/miR-21	Down	79
Neuregulins	Growth factor	Reduce Ser^67^ phosphorylation	Up	80
Exosomes	Extracellular vesicle	miR-21–3'-UTR of PDCD4	Down	82
AC1MMYR2	miRNA inhibitor	miR-21	Up	83

**Table 2:** PDCD4 expression in different subtypes of breast cancer.

Cell line/Tissue Name	Subtype	Technique	Up/Down-regulation	Ref No.
Pregnancy-associated breast cancer	N/A	RT-PCR/IHC	Downregulate	7
IDC	N/A	IHC	Upregulate in cytoplasma and Downregulate in nucleus	8
LET-R / LTEDaro	LuminalA	RT-PCR/IHC	Downregulate	9
HER2aro	Her2 (+)	RT-PCR/IHC	Downregulate	9
DCIS/IDC	N/A	IHC	Downregulate in IDC but slightly decreased in DCIS	29
ER(+) breast cancer tissure	N/A	Reverse phase protein arrays	Downregulation was associated with node positivity	31
MDA-MB-231	TNBC	RT-PCR/IHC	Downregulation	51, 52
HCC1937	TNBC	RT-PCR	Downregulation	59
MDA-MB-468	TNBC	RT-PCR	Downregulation	59
T47D	LuminalA	Western Blot	Downregulation	25

## PDCD4 and Drug Resistance in Breast Cancer

Although various therapeutic agents such as tamoxifen, docetaxel, cisplatin, carboplatin, doxorubicin, gemcitabine, and mitoxantrone can improve overall survival and quality of life to some extent, patients eventually develop resistance to these treatments. The 5-year survival rate for patients with stage IV breast cancer is still as low as 20% (93). Thus, it is urgent to find new approaches that reduce drug resistance and improve the effectiveness of chemotherapy by understanding the relevant mechanisms of drug resistance in breast cancer. Increasing evidence suggests that up-regulation of PDCD4 in breast cancer may improve the sensitivity to systemic therapeutic drugs, especially for patients given neoadjuvant chemotherapy (preoperative chemotherapy where radical surgery is feasible), and thereby reduce tumor size, increase the chance of breast-conserving surgery and prolong lifespan.

### PDCD4 and Resistance toEndocrine Therapy

Estrogens bind to ERs in the nucleus and promote their association with specific estrogen-response elements in the promoter region of target genes. This classic mechanism regulates the development of normal breast tissue and the progression of cancer cells (94). Based on the above mechanism, there are two main strategies to treat breast cancer with endocrine drugs (1): reduce the production of estrogen in the body; (2) competitively inhibit or destroy ERs. There are two main types of ERs in humans that have distinct functions: ERα and ERβ (95). ERα is expressed predominantly as a 66-kD transcript in breast cancer. ERβ has structural homology to ERα, particularly in their DNA-binding domains (95% amino acid identity) and in their ligand-binding domains (55% amino acid identity) (96). ERα is an important marker for breast cancer treatment as altered ERα signaling may cause resistance to endocrine therapy (97). By contrast, ERβ is proposed to inhibit ERα activity and may impair breast cancer cell proliferation by repressing the activation of the MAPK and PI3K signaling pathways (98, 99). ERβ agonists such as LY500307, ERB-041 and WAY200070 efficiently inhibit breast cancer cell growth and invasion. ERβ is now an interesting therapeutic target for patients with triple-negative breast cancer, who lack expression of ERα (100).

About 60%–70% of patients with breast cancer have ER-positive tumors (101). Therefore, inhibition of ER activity is the first therapeutic approach applied for patients with ER-positive breast cancer. Tamoxifen (TAM), a selective estrogen receptor modulator (SERM), is the most common drug for the treatment of ER-positive cancer (102). Nevertheless, approximately 40% of initially ERα-positive breast tumors become resistant to tamoxifen and other endocrine treatments (103). The resistance of ER antagonists may be due to: [1] overexpression of epidermal growth factor receptor (EGFR) and/or the oncogene HER2/neu/ErbB2 (104); [2] splice variants or point mutations in ER (105); [3] alterations in nuclear levels of ER coactivator or corepressor proteins, such as increased coactivator amplified in breast cancer 1 (AIB1) (106) or decreased corepressor nuclear receptor corepressor 1 (NCOR1) gene (107); [4] activation of the MAPK and PI3K/AKT signaling pathways (108, 109); and [5] increased expression of miR-221/222, which downregulate ER post-transcriptionally (110).

PDCD4 enhances sensitivity to several endocrine therapeutic drugs. Colburn and colleagues showed that down-regulation of PDCD4 by antisense RNA significantly reduced chemosensitivity to TAM in MCF-7 cells (111). In addition, PDCD4 is an independent predictive marker for resistance to TAM therapy in recurrent ER-positive breast cancer (112, 113). However, the mechanism through which PDCD4 enhances chemosensitivity to SERMs in breast cancer remains unclear.

Aromatase inhibitors (AIs) such as letrozole, anastrozole, and exemestane are used as first-line treatments for patients with post-menopausal breast cancer and block the peripheral conversion of androgens into estrogens and thereby reduce estrogen levels. Aromatase is the rate-limited enzyme for estrogen biosynthesis. As breast and ovarian cancer cells require estrogen to grow, AIs are administered to either block the production of estrogen or block the action of estrogen on receptors. Unfortunately, resistance to AI treatment is a significant clinical problem for a considerable number of patients with ER-positive breast cancer (114). Chen and his colleagues found that PDCD4 expression was down-regulated in AI-resistant breast cancer cells, and this down-regulation significantly correlated with activation of HER2 signaling in ER-positive breast tumors. HER2 downregulates PDCD4 expression by activating MAPK, AKT, and miR-21 in AI-resistant breast cancer cells. Overexpression of PDCD4 re-sensitizes AI-resistant breast cancer cells to AI, which suggests the potential role of PDCD4 in AI-resistant breast cancer (9).

### PDCD4 and Chemoresistance

PDCD4 is also associated with paclitaxel and doxorubicin resistance in breast cancer (115). Bourguignon et al. found that PDCD4 expression is down-regulated when MCF-7 breast cancer cells are treated with doxorubicin or paclitaxel (116). De Mattos et al. also reported that PDCD4 silencing by siRNAs enhances the resistance of SKBR3 breast cancer cells to paclitaxel and doxorubicin (117). In addition, it was suggested that the hyaluronan-CD44 interaction induced miR-21 production, and the consequent down-regulation of PDCD4 decreased apoptosis and increased the survival of MCF-7 cells and mediated resistance to both paclitaxel and doxorubicin treatment (116). Moreover, recent studies indicated that the miRNA/PDCD4 axis could modulate chemosensitivity in multiple resistant cancers (118–122). Taken together, this suggests that modulation of this axis may be an effective strategy for overcoming chemoresistance in breast cancer. **Table 3** summarizes the relationship between PDCD4 and drug resistance by breast cancer as well as the possible related mechanisms.

**Table 3:** PDCD4 as a drug resistance predictive marker.

Drug Name	Type	Mechanism	Ref No.
Tamoxifen	SERM	Unclear	106-108
Letrozole/Anastrozole/Exemestane	AI	HER2 activate MAPK, AKT and miR-21 pathway	10
Paclitaxel	Phytochemical	Hyaluronan-CD44 interaction induced miR-21 expression	110-112
Doxorubicin	Antibiotic	Hyaluronan-CD44 interaction induced miR-21 expression	110-112

## PDCD4 as a Prognostic Marker in Breast Cancer

A meta-analysis from 23 studies showed that down-regulation of PDCD4 was significantly associated with short overall survival of patients with breast cancer (123). Immunohistochemical staining showed that patients with nuclear PDCD4-positive (NPDCD4-positive) LumB-like tumors had better overall and disease-free survival rates compared to those with NPDCD4-positive LumA-like tumors. By contrast, loss of NPDCD4 expression increased the risk of disease recurrence and death in patients with LumB-like tumors (124). These findings suggest that PDCD4 may be an independent prognostic marker for breast cancer.

## Discussion

PDCD4 is an important suppressor of breast cancer tumorigenesis. Loss of PDCD4 promotes tumor cells proliferation, migration and invasion, and is associated with lymph node metastasis and worse disease-free survival in patients with ER- and HER2-positive breast cancer. This implicates PDCD4 as a possible prognostic marker. Moreover, PDCD4 has been shown to increase the sensitivity of breast cancer cells to endocrine therapy and chemotherapy, which render it as a relevant therapeutic target to overcome chemo-resistance. Thus, compounds that elevate PDCD4 expression are valuable agents for the combination of endocrine therapies or chemotherapies for breast cancer.

Taken together, the future research hotspot for the roles of PDCD4 in breast cancer will likely revolve around the following aspects: [1] the function PDCD4 as a biomarker for breast cancer diagnosis, prognosis, and drug-resistance; [2] the identification of novel upstream regulators and downstream target genes of PDCD4 and molecular biological mechanisms; [3] the development of small molecular and targeted drugs related to PDCD4.

## Author Contributions

JZ contributed to conception and design of the review. JZ and YCL organized the tables and the figure. QC wrote the first draft of the manuscript. HSY wrote one section (PDCD4 function) of the manuscript. All authors contributed to the article and approved the submitted version.

## Funding

This work was supported by grant from the National Natural Science Foundation of China (No. 81402192).

## Conflict of Interest

The authors declare that the research was conducted in the absence of any commercial or financial relationships that could be construed as a potential conflict of interest.

## Publisher’s Note

All claims expressed in this article are solely those of the authors and do not necessarily represent those of their affiliated organizations, or those of the publisher, the editors and the reviewers. Any product that may be evaluated in this article, or claim that may be made by its manufacturer, is not guaranteed or endorsed by the publisher.
